# Atherosclerotic Cardiovascular Disease and Cancer

**DOI:** 10.1111/imr.70141

**Published:** 2026-07-12

**Authors:** Anaïs Amend, Hauke Horstmann, Kory J. Lavine, Chiara Giannarelli, Kathryn J. Moore

**Affiliations:** ^1^ Leon H. Charney Division of Cardiology, Department of Medicine NYU Grossman School of Medicine New York New York USA; ^2^ Cardiovascular Research Center NYU Langone Health New York New York USA; ^3^ Division of Cardiology, Center for Cardiovascular Research, Department of Medicine Washington University School of Medicine Saint Louis Missouri USA; ^4^ Department of Pathology and Immunology Washington University School of Medicine Saint Louis Missouri USA; ^5^ Department of Pathology NYU Grossman School of Medicine New York New York USA; ^6^ Department of Cell Biology NYU Grossman School of Medicine New York New York USA

**Keywords:** atherosclerosis, cardio‐oncology, clonal hematopoiesis, extracellular vesicles, immune checkpoints, reverse cardio‐oncology, trained immunity

## Abstract

Atherosclerotic cardiovascular disease (ASCVD) and cancer are increasingly recognized as interconnected diseases linked by shared immune mechanisms rather than merely overlapping risk factors. Common exposures such as smoking, obesity, diabetes, and dyslipidemia, together with aging and clonal hematopoiesis of indeterminate potential (CHIP), establish a chronic inflammatory milieu that drives both pathologies through coordinated reprogramming of myeloid and lymphoid compartments. Within this framework, a forward cardio‐oncology axis is increasingly recognized, in which cancer therapies including chemotherapies, radiation, and immune checkpoint inhibitors induce cardiovascular injury, manifesting as cardiomyopathy, accelerated atherosclerosis, and immune‐mediated myocarditis. Complementing this, a reverse axis has emerged in which cardiovascular injury states such as myocardial infarction, ischemia, and heart failure actively promote cancer initiation and progression through hematopoietic remodeling, extracellular vesicle‐mediated communication, cardiac‐derived factors, and immunosuppressive myeloid bias. At the tissue level, immune checkpoint pathways including PD‐1, PD‐L1, CTLA‐4, LAG‐3, and TIM‐3 form spatially organized regulatory networks within atherosclerotic plaques. Their therapeutic perturbation restores T cell activity but may disrupt local immune homeostasis and promote plaque instability. In parallel, inflammatory cytokines such as IL‐1β, IL‐6, and TNF‐α, often amplified by CHIP‐associated clones, provide a mechanistic bridge linking atherogenesis with tumor immune evasion. Together, these observations support a unified view of ASCVD and cancer as immune‐driven diseases connected by bidirectional axes of interaction. This review integrates emerging mechanistic and clinical evidence and outlines how immune‐based stratification and targeted modulation of inflammation may enable more precise management of patients at the intersection of cardiovascular disease and cancer.

## 1 Introduction

Atherosclerotic cardiovascular disease (ASCVD) and cancer are the two leading causes of death globally and are increasingly understood as mechanistically interconnected conditions rather than parallel disease entities. A broad spectrum of shared exposures, including smoking, obesity, physical inactivity, diabetes, dyslipidemia, and unhealthy diet, converge on a chronic inflammatory milieu that fuels both pathologies [1, 2]. Beyond these common risk factors, aging‐associated processes such as clonal hematopoiesis of indeterminate potential (CHIP) further amplify systemic inflammation and reshape hematopoietic output, reinforcing a shared biological substrate that links cardiovascular disease and malignancy [3, 4, 5].

Within this framework, a bidirectional model has emerged that distinguishes two interacting trajectories. A forward trajectory captures the impact of cancer therapies on the cardiovascular system, where cytotoxic, targeted, and immune‐based treatments induce vascular injury, myocardial dysfunction, and immune‐mediated cardiac inflammation [6, 7]. A complementary reverse trajectory reflects the capacity of cardiovascular injury states, including myocardial infarction, ischemia, and heart failure, to reprogram systemic immunity in ways that promote tumor initiation and progression. The frequent co‐occurrence of ASCVD and cancer therefore reflects not only shared risk exposures but also active biological crosstalk between the two disease states [8].

The clinical and mechanistic intersection of these diseases has become particularly evident with the introduction of immune checkpoint inhibitors. Early observations of immune checkpoint inhibitor‐related myocarditis, particularly following combined PD‐1 and CTLA‐4 blockade, demonstrated that reinvigoration of T cell responses can precipitate severe cardiac autoimmunity, likely through recognition of shared antigens between tumor and cardiac tissue [9, 10]. Subsequent work has expanded the spectrum of cardiovascular complications associated with cancer therapies, encompassing vascular dysfunction, arrhythmias, and myocardial injury, thereby defining a distinct clinical and biological interface between oncology and cardiovascular medicine [7, 11].

At the mechanistic level, innate and adaptive immune pathways form a shared axis of disease propagation. Myeloid cell reprogramming, T cell dysfunction, and cytokine‐driven inflammation integrate signals from metabolic stress, tissue injury, and tumor‐derived factors. Neutrophil extracellular traps (NETs) exemplify this bidirectional interplay, linking tumor‐associated inflammation to vascular thrombosis while simultaneously facilitating metastatic dissemination [12, 13, 14].

Together, these observations support a unified view of ASCVD and cancer as immune‐driven diseases connected through shared inflammatory circuits and bidirectional systemic communication. Dissecting these interactions provides a framework for understanding disease co‐occurrence and progression and highlights the need for integrated strategies that address both cardiovascular and oncologic risk through targeted modulation of immune pathways.

## 2 Converging Risk Factors in ASCVD and Cancer

### 2.1 Shared Modifiable Risk Factors

**FIGURE 1:**
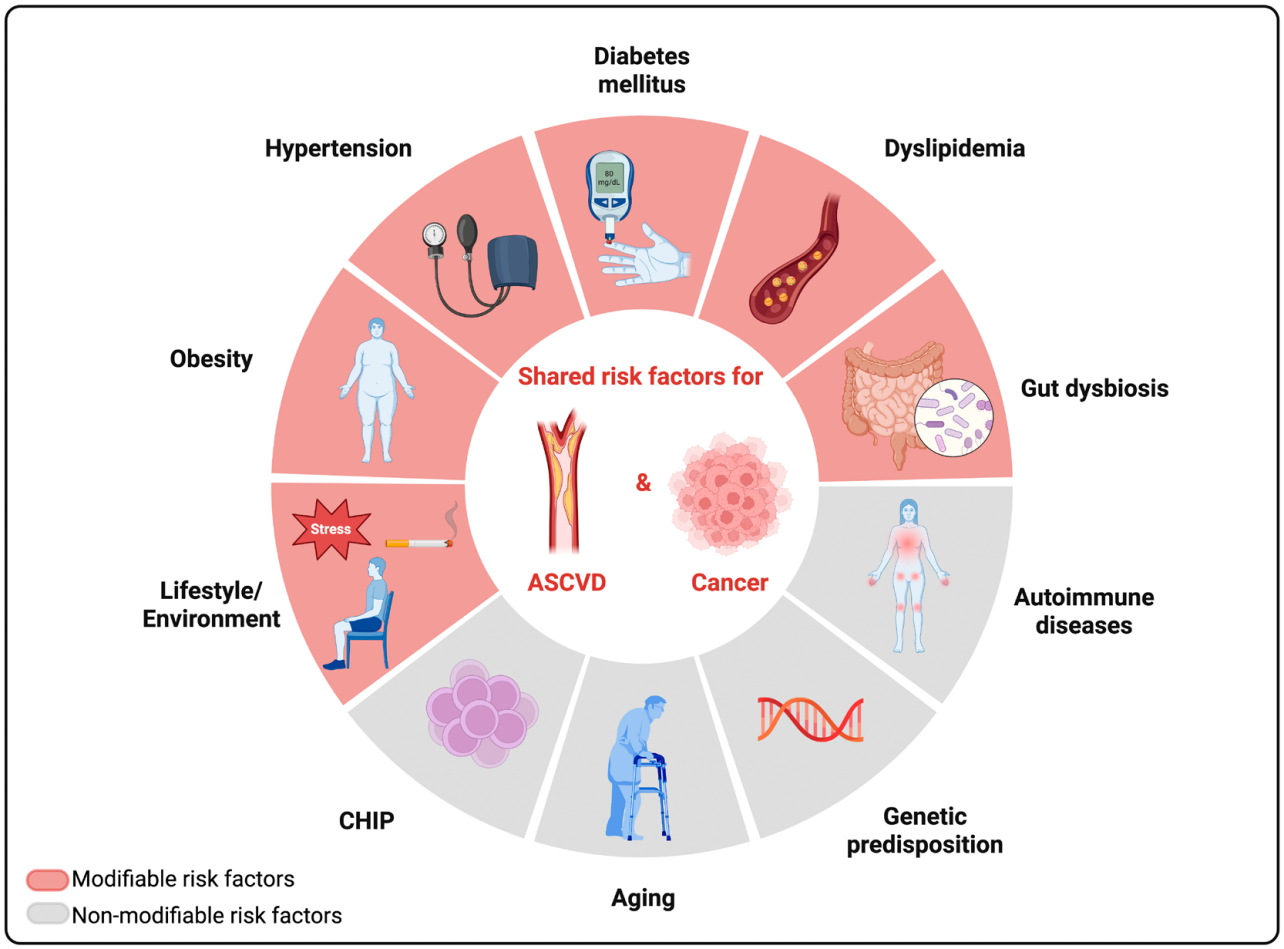
Shared risk factors linking atherosclerotic cardiovascular disease and cancer. Atherosclerotic cardiovascular disease (ASCVD) and cancer share numerous overlapping biological, environmental, and clinical risk factors that contribute to the bidirectional relationship observed in cardio‐oncology. Modifiable risk factors include obesity, diabetes mellitus, hypertension, dyslipidemia, gut dysbiosis, and adverse lifestyle or environmental exposures such as smoking, psychosocial stress, physical inactivity, and poor diet. Non‐modifiable contributors include clonal hematopoiesis of indeterminate potential (CHIP), aging, genetic predisposition, and autoimmune disease. Together, these shared determinants promote chronic inflammation, immune dysregulation, metabolic dysfunction, and tissue remodeling, creating a common pathogenic framework that increases susceptibility to both cardiovascular disease and malignancy. The convergence of these pathways highlights the need for integrated prevention and therapeutic strategies across cardiovascular and oncologic care.

Obesity, diabetes mellitus, dyslipidemia, and hypertension are the core components of the metabolic syndrome. Alongside smoking and physical inactivity, they represent well‐established shared modifiable risk factors for both ASCVD and cancer (Figure 1) [1, 2, 15]. At the molecular level, each of these factors drives atherogenesis and oncogenesis through overlapping biological mechanisms, including chronic inflammation and immune dysregulation [15].

In obesity, the expansion of visceral adipose tissue induces chronic low‐grade inflammation, characterized by elevated circulating levels of interleukin (IL)‐1β, IL‐6, and tumor necrosis factor (TNF)‐α [16, 17, 18, 19]. This inflammatory state promotes endothelial dysfunction, initiating atherosclerotic plaque formation [20, 21]. In parallel, it drives pro‐tumorigenic macrophage polarization within the tumor microenvironment [22, 23]. Adipokine dysregulation amplifies these effects: increased leptin and reduced adiponectin enhance cancer cell proliferation, survival signaling, and tumor angiogenesis [23]. Epidemiologically, an increase in BMI is associated with a higher risk of 10 of 22 common cancers in a cohort of 5.24 million adults [24]. Physical inactivity independently compounds this burden by further driving visceral fat accumulation and activating a network of inflammatory pathways that converge in atherogenesis and tumor growth [25].

Type 2 diabetes mellitus often develops in the context of obesity‐associated insulin resistance [26, 27, 28], and increases disease risk through complementary mechanisms. Chronic hyperinsulinemia activates Insulin‐like growth factor 1 (IGF‐1) receptor signaling, leading to the activation of PI3K/AKT and RAS/MAPK pathways [29]. These pathways promote smooth muscle cell proliferation in plaques, while also driving tumor cell growth, survival, and resistance to apoptosis [30, 31]. The insulin/IGF‐1 axis therefore represents a shared growth‐promoting pathway. Chronic hyperglycemia fuels these effects by promoting advanced glycation end‐product (AGE) accumulation and signaling through their receptor RAGE. This triggers oxidative stress and endothelial inflammation, processes that accelerate plaque progression [32, 33]. In cancer, the same AGE–RAGE axis reprograms oncogenic signaling and sustains a pro‐tumorigenic inflammatory environment [34, 35].

Dyslipidemia contributes an additional shared mechanistic layer: oxidized low‐density lipoprotein (LDL) undergoes scavenger receptor‐mediated uptake by macrophages, driving foam cell formation and atherosclerotic plaque progression [36]. In cancer, cholesterol‐enriched lipid rafts act as dynamic signaling platforms that concentrate and activate key oncogenic pathways, driving tumor cell growth, adhesion, migration, invasion, and resistance to apoptosis [37, 38]. Thus, lipid excess links plaque lipid accumulation with membrane‐dependent oncogenic signaling.

Hypertension bridges cardiovascular and cancer pathology through angiotensin II (Ang II). Via AT1 receptor activation, Ang II stimulates NADPH oxidase, increasing vascular superoxide production, depleting nitric oxide bioavailability, and driving oxidative endothelial injury that accelerates atherogenesis [39, 40, 41, 42]. In parallel, Ang II induces VEGF transcription via PKC–NF‐κB and HIF‐1α pathways, promoting tumor angiogenesis and cancer progression [43, 44, 45].

Smoking amplifies both vascular and oncogenic injury through converging toxicant‐driven mechanisms. Cigarette smoke delivers a complex mixture of reactive oxygen species and free radicals that overwhelm antioxidant defenses, leading to endothelial dysfunction, lipid oxidation, and the formation of foam cells that drive atherogenesis [46] and can promote DNA damage [47]. The major tobacco carcinogens, polycyclic aromatic hydrocarbons and tobacco‐specific nitrosamines, operate as shared pathological agents. For example, benzo[a]pyrene forms DNA adducts that induce TP53 mutations leading to cancer [48], while simultaneously exacerbating atherosclerosis via macrophage inflammation and lipid dysregulation [49]. Nicotine reinforces this dual pathology by acting through non‐neuronal nicotinic acetylcholine receptors to simultaneously accelerate plaque neovascularization and tumor growth, potentially via upregulation of VEGF, nitric oxide, and prostacyclin [50]. Smoking therefore couples genotoxic injury with vascular inflammation and angiogenic remodeling.

Psychosocial stress is a further common modifiable risk factor acting through a shared neuroendocrine axis. Chronic activation of the hypothalamic–pituitary–adrenal axis and sympathetic nervous system drives sustained cortisol and catecholamine excess, elevating blood pressure, disrupting endothelial vasodilatory balance, and promoting a pro‐inflammatory and procoagulant state through cytokine upregulation, platelet activation, and increased fibrinogen, collectively accelerating atherosclerotic progression [51]. Hyperactivity of the amygdala, a brain region involved in stress, independently predicted future cardiovascular events in this longitudinal cohort study, with the relationship mediated through increased bone marrow activity and arterial inflammation [52]. The same catecholamine surge activates β‐adrenergic receptors on tumor and stromal cells, engaging PKA/EPAC pathways that drive angiogenesis, immune evasion, and metastasis [53]. A meta‐analysis of 165 studies reinforces that psychosocial stress may be associated with increased cancer incidence and worse survival [54].

Emerging evidence highlights gut dysbiosis as a potential upstream regulator of both ASCVD and cancer. Microbial metabolism of dietary phosphatidylcholine and L‐carnitine generates trimethylamine N‐oxide (TMAO), which accelerates atherosclerosis by upregulating macrophage scavenger receptors SR‐A and CD36 to enhance foam cell formation [55], and enhances platelet reactivity through augmented intracellular calcium signaling [56]. Beyond its cardiovascular effects, TMAO may promote tumor cell proliferation, angiogenesis, and invasion in colorectal cancer and hepatocellular carcinoma through activation of respectively Wnt/β‐catenin and MAPK signaling, NLRP3 inflammasome induction, and inhibition of FXR‐mediated tumor suppression [57]. The gut microbiome may therefore represent a modifiable metabolic and immunological interface linking diet, vascular inflammation, thrombosis, and cancer progression.

### 2.2 Clonal Hematopoiesis of Indeterminate Potential as a Unifying Molecular Driver

Both ACVD and cancer share genetic predisposition [58] and aging as common risk factors [1, 32]. Aging in particular underlies the emergence of CHIP, defined by acquisition of somatic mutations in hematopoietic stem and progenitor cells that confer clonal proliferative advantage without overt hematologic malignancy [3], with DNMT3A, TET2, ASXL1, and JAK2 representing the most commonly affected genes [59]. CHIP affects approximately 10% of individuals aged 70–79 years and nearly 20% of nonagenarians in unselected population cohorts [4]. DNMT3A and TET2 mutations collectively account for the majority of clonal variants across all age strata [60]; when ultra‐sensitive sequencing of broader gene panels is applied, detectable clonal hematopoiesis is present in the majority (> 60%) of octogenarians [61]. On the cardiovascular side, CHIP carriers face significantly increased risks of incident coronary heart disease and ischemic stroke, as first established in a landmark exome sequencing study of 17,182 individuals [4], with TET2‐, ASXL1‐, JAK2‐, and DNMT3A‐mutant CHIP subsequently shown to independently nearly double the risk of coronary artery disease [5]. Among patients with solid tumors, CHIP prevalence ranges from 10% to 30% depending on sequencing sensitivity and cohort age composition, with DNMT3A the most frequently mutated gene and prevalence rising steeply with age [62, 63]; in breast cancer specifically, CHIP is detectable in ~15% of patients at diagnosis, and cytotoxic chemotherapy selectively expands small TP53‐ and PPM1D‐mutant clones that carry elevated risk of progression to treatment‐related myeloid neoplasia [63]. One mechanism resides in NLRP3 inflammasome hyperactivation and IL‐1β hypersecretion by TET2‐mutant macrophages that accelerate atherosclerotic plaque formation, an effect abrogated by NLRP3 inhibition in murine models [64], and restrained by colchicine [65] and IL‐6 receptor blockade [66], establishing multiple tractable therapeutic axes. On the oncologic side, TET2‐, DNMT3A‐, and ASXL1‐mutant clones are recognized precursors to myeloid malignancies, positioning CHIP as a molecular bridge between normal aging and pre‐malignancy [3]. A systematic review and meta‐analysis of 88 cohort studies confirmed that CHIP confers a 40% increased risk of composite cardiovascular events, a 76% increased risk of coronary heart disease, and a 46% increase in cancer‐specific mortality [67]. Critically, these two dimensions converge: in patients with confirmed CAD, CHIP independently predicts mortality [68], and in multiple myeloma patients undergoing hematopoietic cell transplantation, CHIP predicted post‐transplant cardiovascular events, demonstrating the CHIP‐CVD‐cancer triad in an oncologic population [69]. CHIP has consequently emerged as a pan‐disease aging biomarker connecting inflammaging to both atherosclerosis and malignancy, with pharmacological strategies targeting NLRP3, JAK, and epigenetic regulators offering potential to simultaneously attenuate cardiovascular and oncologic risk [70, 71].

### 2.3 Systemic Autoimmunity as a Convergent Risk Factor

Systemic inflammatory and autoimmune diseases constitute an underappreciated but mechanistically coherent class of shared risk factors for ASCVD and cancer. Women with systemic lupus erythematosus (SLE) aged 35–44 carry over a 50‐fold excess myocardial infarction risk relative to age‐matched Framingham Offspring Study controls [72]. A meta‐analysis of 111,758 patients indicates that cardiovascular mortality is increased by 50% in patients with rheumatoid arthritis (RA) [73]. The oncological burden mirrors this vascular risk: cumulative inflammatory activity in RA elevates lymphoma risk dose‐dependently to an odds ratio of 61.6 in the highest disease‐activity decile, with immune dysregulation rather than antirheumatic therapy identified as the primary oncogenic driver [74]. One mechanism driving this process is the formation of cholesterol crystal‐activated neutrophil extracellular traps (NETs), which prime plaque macrophages for NLRP3‐dependent IL‐1β production and amplify Th17‐mediated leukocyte recruitment within atherosclerotic plaques [75] while in the tumor microenvironment NETs scaffold cancer cell migration and pre‐metastatic niche formation [76]. Concurrently, the type I interferon (IFN‐I) signature intrinsic to SLE drives NET release, impairs endothelial repair, and accelerates subclinical atherosclerosis through mechanisms partially attenuated by JAK inhibition [77]. The chronically activated complement system in SLE and RA generates elevated circulating and synovial C3a and C5a [78, 79], which can promote vascular endothelial activation and sustained plaque inflammation within the atheromatous vessel wall [80, 81], while paradoxically co‐opting C3aR and C5aR1 signaling in the tumor microenvironment to suppress anti‐tumor immunity and promote tumor growth [82]. Autoimmune‐driven chronic immune activation thus represents a distinct but synergistic stratum of shared cardiovascular and oncological risk.

## 3 Common Immune Pathways Driving Atherosclerosis and Cancer

### 3.1 Systemic Inflammation as Shared Currency

IL‐1β, IL‐6, TNF‐α, and CCL2 are cardinal cytokines operating across both atherogenesis [83, 84] and tumor promotion [85, 86, 87] through overlapping and mutually amplifying signaling networks. IL‐1β drives endothelial adhesion molecule expression, monocyte recruitment, and NLRP3 inflammasome‐amplified macrophage activation within atherosclerotic plaques [88, 89]. The same pathway enables tumor immune evasion by promoting regulatory T cell expansion, M2‐like macrophage polarization, and NF‐κB‐dependent anti‐apoptotic gene expression in cancer cells [22, 87, 90]. Tumors themselves secrete inflammatory mediators such as IL‐6, TNF‐α, and IL‐1β [87] that contribute to a pro‐atherogenic milieu [91], with elevated CRP independently predicting cardiovascular mortality in cancer patients [92]. IL‐6, produced by stromal cells, tumor‐associated macrophages, and activated endothelium, drives STAT3 signaling that simultaneously sustains atherosclerotic plaque macrophage survival and mediates cancer cell proliferative resistance to therapy [66, 93]. TNF‐α and CCL2 reinforce this shared circuitry: TNF‐α amplifies NF‐κB‐driven cytokine production in foam cells and the tumor microenvironment alike, while CCL2‐directed monocyte trafficking feeds macrophage accumulation in atherosclerotic lesions and sustains the immunosuppressive myeloid infiltrate of solid tumors [94, 95, 96, 97, 98, 99, 100]. These molecular convergences have measurable clinical correlates: residual inflammatory risk, indexed by hsCRP elevation despite statin therapy, predicts cardiovascular events independently of LDL cholesterol, establishing inflammation as a tractable therapeutic target beyond lipid biology [101, 102]. At the hematopoietic level, myocardial infarction triggers emergency mobilization of the splenic monocyte reservoir and accelerated myelopoiesis, generating a systemic inflammatory wave that propagates well beyond the ischemic myocardium and may prime both plaque instability and tumor‐permissive microenvironments [103, 104].

### 3.2 Myeloid Cell Biology: Monocytes, Macrophages, and Neutrophils

Monocytes and macrophages are central cellular executors of both atherosclerosis and cancer‐related immune dysfunction. In ASCVD, classical monocytes, recruited to the arterial wall through CCL2–CCR2 signaling, differentiate into lipid‐laden macrophage foam cells that are the proximate drivers of necrotic core expansion and plaque vulnerability [100, 105, 106]. Defective efferocytosis, the phagocytic clearance of apoptotic cells, is a critical driver of this process: when macrophage corpse‐removal capacity is overwhelmed, apoptotic foam cells undergo secondary necrosis, fueling necrotic core expansion and inflammation that cannot resolve [107, 108]. A parallel but inverted subversion operates in tumors, where tumor‐associated macrophage (TAM)‐mediated efferocytosis of apoptotic cancer cells is co‐opted to generate immune tolerance rather than resolution, with MerTK and Axl receptor signaling skewing macrophages toward an IL‐10‐producing, immunosuppressive phenotype after each round of corpse ingestion [109, 110]. The binary M1/M2 polarization framework has been substantially superseded by single‐cell transcriptomics, which resolves a continuous spectrum of macrophage phenotypes in both atherosclerotic plaques [111, 112, 113, 114, 115] and the tumor microenvironment [116, 117, 118]. Within each of these disease contexts, monocytes differentiate into a wide array of macrophage states.

In atherosclerotic plaques, pro‐inflammatory, lipid‐handling, and resolution‐associated states coexist, which exemplifies simple dichotomous classification. Shared metabolic reprogramming may bridge both disease contexts: suppression of oxidative phosphorylation and upregulation of glycolysis through the HIF‐1α axis have been implicated in pro‐inflammatory macrophage programming and have been proposed as shared therapeutic targets in atherosclerosis and cancer, though direct comparative evidence in human tissue remains limited [119, 120]. For example, CD147 (Basigin) illustrates how a single surface receptor simultaneously enforces M1‐like polarization and compromises efferocytic capacity in atherosclerotic lesions [121]. Competent efferocytosis, by contrast, reprograms macrophage metabolism through apoptotic cell‐derived arginine catabolism: Arginase 1 converts ingested arginine to ornithine, which ornithine decarboxylase further processes to the polyamine putrescine, sustaining subsequent rounds of corpse internalization and driving inflammation resolution in atherosclerosis regression models [122]. A mechanistically analogous program may operate in high‐apoptotic‐burden tumor microenvironments, where TAMs encounter comparably abundant apoptotic cargo and amino acid metabolism similarly modulates their immunomodulatory output [123]. Pan‐cancer single‐cell transcriptomics reveals four recurrent TAM programs: a pro‐inflammatory NLRP3–IL‐1β program and a hypoxia‐driven SPP1–osteopontin program that predominantly support tumor growth, set against antitumor IFN‐I–ISG15 and IFNγ–CXCL9 programs that recruit and license cytotoxic T cells [124, 125]. The ratio of CXCL9^+^ to SPP1^+^ macrophages serves as an integrative prognostic index, with SPP1 polarity tracking hypoxic, angiogenic niches and CXCL9 polarity marking interferon‐activated, immune‐permissive states [126]. This spectrum of context‐dependent states in the tumor microenvironment mirrors the functional heterogeneity increasingly recognized in atherosclerotic plaques, where pro‐inflammatory, lipid‐handling, and resolution‐associated macrophage populations coexist and similarly resist dichotomous classification. Tissue‐context specificity further refines this myeloid landscape: mural cell‐derived CCL2 and macrophage migration inhibitory factor sustain a homeostatic macrophage niche within the atherosclerotic plaque, positioning macrophages in viable fibrous cap regions and preserving efferocytic capacity [127]. Disruption of this axis redirects macrophages toward the necrotic core and accelerates atheroprogression, illustrating how the same chemokines considered detrimental during monocyte recruitment exert atheroprotective effects on mature tissue‐resident macrophages [127].

Neutrophils constitute an underappreciated cellular nexus between atherosclerosis and cancer; however, they are systematically absent from most single‐cell transcriptomic datasets. Their low RNA content, high intracellular RNase activity, and sensitivity to post‐collection degradation reduce the number of detectable transcripts per cell, leading them to be discarded by standard quality‐control filters. Where neutrophils have been specifically captured and profiled, scRNA‐seq resolves conserved transcriptional states across patients and tumor types [118, 128, 129, 130]. Tumor‐associated neutrophils exhibit marked plasticity. In early carcinogenesis, IFN‐β primes an N1‐like antitumor state: these cells generate cytotoxic hydrogen peroxide, upregulate TNF and ICAM1, and can acquire antigen‐presenting features that amplify CD8^+^ T cell responses [131, 132, 133]. As tumors progress, TGF‐β drives a shift toward an N2‐like pro‐tumor phenotype characterized by arginase 1 upregulation, depleting local arginine to suppress T cell activation, alongside MMP9‐mediated angiogenesis, prostaglandin E_2_‐driven immunosuppression, and surface expression of immune checkpoint ligands PD‐L1 and VISTA [87, 131, 134, 135]. In atherosclerotic plaques, neutrophil‐derived myeloperoxidase and elastase oxidize LDL and degrade the fibrous cap [14, 136]. Neutrophils also extrude NETs—decondensed chromatin scaffolds decorated with citrullinated histone H3, myeloperoxidase, and elastase [137, 138]. NETs accumulate in rupture‐prone regions and drive inflammasome activation, plaque progression, and atherothrombosis [13, 75, 139]. NETs additionally function as bidirectional mediators of the CVD–cancer axis: cancer‐associated NETosis drives thrombotic risk in cancer patients [140, 141], while the same pro‐inflammatory milieu that promotes plaque NETosis may amplify cancer progression. NETs in turn remodel the pre‐metastatic niche, expand immunosuppressive cell populations, and impair anti‐tumor cytotoxic T lymphocyte responses [12, 142, 143].

### 3.3 Trained Immunity: Epigenetic Amplification of Shared Inflammation

Trained immunity is a form of innate immune memory mediated by epigenetic reprogramming of hematopoietic stem and progenitor cells (HSPCs) and their progeny. It amplifies inflammatory responses to diverse stimuli and constitutes a fundamental mechanism linking cardiovascular risk factors to sustained systemic inflammation [144]. Atherosclerosis‐promoting factors, including oxidized LDL, hyperglycemia, and IL‐1β, induce H3K4me3 and H3K27ac marks at the promoters of innate immune response genes in HSPCs and macrophages [145, 146]. This epigenetic remodeling generates augmented cytokine production upon rechallenge, a memory that persists for weeks to months after the initial stimulus [147]. Trained immunity inhibitors including metformin and rapamycin could dampen maladaptive innate immune amplification in atherosclerosis [148]. The mTOR–HIF‐1α–glycolysis axis is a key metabolic effector of this reprogramming [149]. However, when trained immunity operates chronically, this initial adaptive program turns maladaptive. Rather than resolving after the initial stimulus, persistent inflammatory conditioning drives durable myeloid‐biased hematopoietic output that can sustain both vascular inflammation and tumor‐promoting immune suppression [150]. Although targeting the mTOR–HIF‐1α–glycolysis axis has been explored for targeting maladaptive trained immunity, the effect of metabolic interventions acting on this pathway are highly context dependent, and these are not directly translatable across disease contexts [149].

When this inflammatory conditioning operates chronically, a parallel shift in hematopoietic output emerges: sustained signaling through inflammatory cytokines and myeloid growth factors promotes myeloid‐biased differentiation of hematopoietic stem cells (HSCs), expanding the pool of circulating inflammatory myeloid cells that can sustain both vascular inflammation and potentially tumor‐promoting immune suppression [151, 152, 153, 154]. This myeloid‐biased output expands progressively with age and chronic inflammation at the expense of balanced progenitors that sustain lymphoid output [155]. In humans, aged HSCs increase in frequency and become less quiescent. They transcriptionally upregulate genes associated with myeloid lineage specification and myeloid malignancy—a pattern conserved across species and evident in immunophenotypically normal bone marrow [156]. Functionally, this shift reduces naïve T and B cell generation and elevates circulating IL‐1α and IL‐1β. Targeted depletion of myeloid‐biased hematopoeietic stem cells in aged mice partially restores naïve T and B cell generation, lowers circulating IL‐1α, and improves adaptive immune responses, demonstrating that hematopoietic composition is a tractable therapeutic target [157]. The mechanisms driving myeloid expansion are additive. Population‐dynamics modeling of NFκB‐dysregulated HSPCs reveals two parallel processes: reduced lymphoid‐fate specification at the multipotent progenitor stage, and enhanced net proliferation of early myeloid‐primed progenitors. Crucially, this program is fully reproduced when wild‐type HSPCs are transplanted into an inflamed niche, establishing cell‐extrinsic inflammatory signals as sufficient drivers without HSC‐intrinsic mutations [158]. IL‐1β, abundant in both atherosclerotic plaques and the tumor microenvironment, is a key upstream inducer of this bias [159]. This creates a feed‐forward loop in which myeloid‐skewed output sustains the inflammatory milieu that drives further progenitor distortion, linking trained myelopoiesis to the shared inflammatory substrate of cardiovascular disease and cancer.

This feed‐forward dynamic is not unique to chronic sterile inflammation. The canonical trained immunity stimuli, β‐glucan and BCG vaccination, establish the same principle at the HSPC level: both directly skew progenitor differentiation toward myelopoiesis, generating monocytes and granulocytes with augmented innate effector capacity [160, 161, 162]. Physiologically, this myeloid amplification is adaptive and transient. Chronic inflammation converts it into a self‐sustaining pathological program. Epigenetically rewired HSPCs continuously replenish a myeloid output with heightened inflammatory and immunosuppressive capacity [150, 163]. MI‐driven emergency myelopoiesis is the archetypal cardiovascular trigger of this program: trained immunity signatures installed at the HSPC level persist beyond the acute ischemic event, driving myeloid‐derived suppressor cell expansion within the tumor microenvironment, attenuating CD8^+^ T cell‐mediated anti‐tumor surveillance, and sustaining a niche permissive to tumor growth [164]. Ischemia across multiple vascular beds drives this same hematopoietic reprogramming and promotes mammary tumor growth in murine models [165], establishing a shared HSPC‐level mechanism through which cardiovascular injury and tumor‐promoting immunity are coupled.

### 3.4 Adaptive Immune Reprogramming: T Cells and the Exhaustion Axis

In both atherosclerotic plaques and the tumor microenvironment, T cells are clonally expanded, antigen‐experienced participants shaped by chronic stimulation into functionally heterogeneous states. High‐dimensional single‐cell profiling has defined this landscape in granular detail, revealing shared exhaustion programs and adaptive immune microarchitectures across both disease contexts [111, 112, 113, 115, 166, 167, 168]. In atherosclerosis, oxidized ApoB‐containing lipoproteins act as candidate neo‐self‐antigens [169, 170]; plaque‐derived dendritic cells process these antigens and migrate to regional lymph nodes, priming antigen‐specific T cells via MHC class I and II pathways [83, 171, 172], in a manner mechanistically analogous to neoantigen presentation in tumor‐draining lymph nodes. scRNA‐seq with TCR sequencing of human carotid plaques identifies plaque‐specific clonal expansion of activated CD4^+^ T cells with foam cell interaction signatures, supporting antigen‐driven local immune activation in atherosclerosis [173]. The CD4^+^ Th1 subset is the dominant effector in both contexts: in ASCVD, Th1 cells drive plaque progression through IFN‐γ [174, 175]; in the tumor microenvironment (TME), the Th1–IFN‐γ axis sustains cytotoxic CD8^+^ T cell responses and represents a key correlate of anti‐tumor immunity and ICI responsiveness. This functional overlap defines a central cardiovascular liability of ICI therapy, whereby therapeutic amplification of Th1 activity that enhances tumor control concurrently accelerates atherogenic inflammation. Critically, chronic IFN‐γ signaling in the TME induces PD‐L1 and IDO expression on tumor and stromal cells via adaptive immune resistance [176], directly fueling the CD8^+^ T cell exhaustion program detailed in the following section and linking Th1 effector output to progressive immune tolerance in both microenvironments.

FOXP3^+^ Tregs counterbalance Th1‐driven inflammation in both settings, exerting plaque‐stabilizing effects in ASCVD and constituting a dominant immunosuppressive population in the TME, where they are recruited by tumor‐derived CCL22 and maintained by TGF‐β. Their role in symptomatic atherosclerotic disease remains debated: circulating Treg numbers decline in acute coronary presentations [177], and plaque‐resident apoB‐reactive Tregs progressively lose FoxP3 expression as atherosclerosis advances, transitioning into pathogenic Th1 and Th17 effectors [169]. Comparable Treg instability under chronic inflammatory pressure in the TME promotes Th17 programs, which drive IL‐17A‐mediated angiogenesis and immunosuppressive neutrophil recruitment in cancer. Th17 responses are further amplified by ICI therapy and are particularly prominent in immune‐related enterocolitis, though irAE immunopathology is organ‐specific, with Th1–IFN‐γ programs predominating in dermatitis and other tissue toxicities [178]. This shared reliance on Treg‐mediated control creates a convergent vulnerability, as disruption of Treg function by ICI therapy destabilizes plaque homeostasis while simultaneously altering tumor immune evasion.

Progressive CD8^+^ T cell exhaustion is the common endpoint of chronic antigen exposure in both microenvironments. It is marked by co‐expression of PD‐1, TIM‐3, LAG‐3, TIGIT, and the transcription factor TOX, which together define key molecular targets of ICI therapy [179, 180, 181]. Exhaustion is a continuum rather than a binary state: TCF1^+^ progenitor‐exhausted cells retain proliferative capacity and are the primary responders to PD‐1 blockade, while terminally exhausted TCF1^−^ cells bear TOX‐driven epigenetic entrenchment of dysfunction that is largely irreversible [182]. High‐dimensional profiling of human atherosclerotic plaques reveals that advanced lesions harbor substantial populations of PD‐1^+^ LAG‐3^+^ TIM‐3^+^ CD8^+^ T cells transcriptionally resembling tumor infiltrating lymphocyte exhaustion states [113], suggesting that the TCF1^+^ progenitor pool within plaques may be similarly reinvigorated and destabilized by ICI therapy. Tertiary lymphoid structures (TLS) amplify this antigen‐driven program in both contexts. Adventitia‐associated lymphoid organs (ATLOs) sustain local T cell priming and germinal center‐like B cell responses adjacent to atherosclerotic lesions [183, 184, 185], while tumor‐associated TLS predict ICI response and overall survival across cancer types, with high TLS density correlating with enhanced CD8^+^ T cell infiltration and local antibody production [186]. This convergence of structural organization and molecular programming, encompassing shared exhaustion signatures, checkpoint receptor expression, and lymphoid architecture, provides the immunological basis for the cardiovascular effects of ICI therapy.

## 4 Immune Checkpoints at the Intersection of Cancer Therapy and ASCVD

### 4.1 The Checkpoint Landscape of Human Atherosclerotic Plaques

**FIGURE 2:**
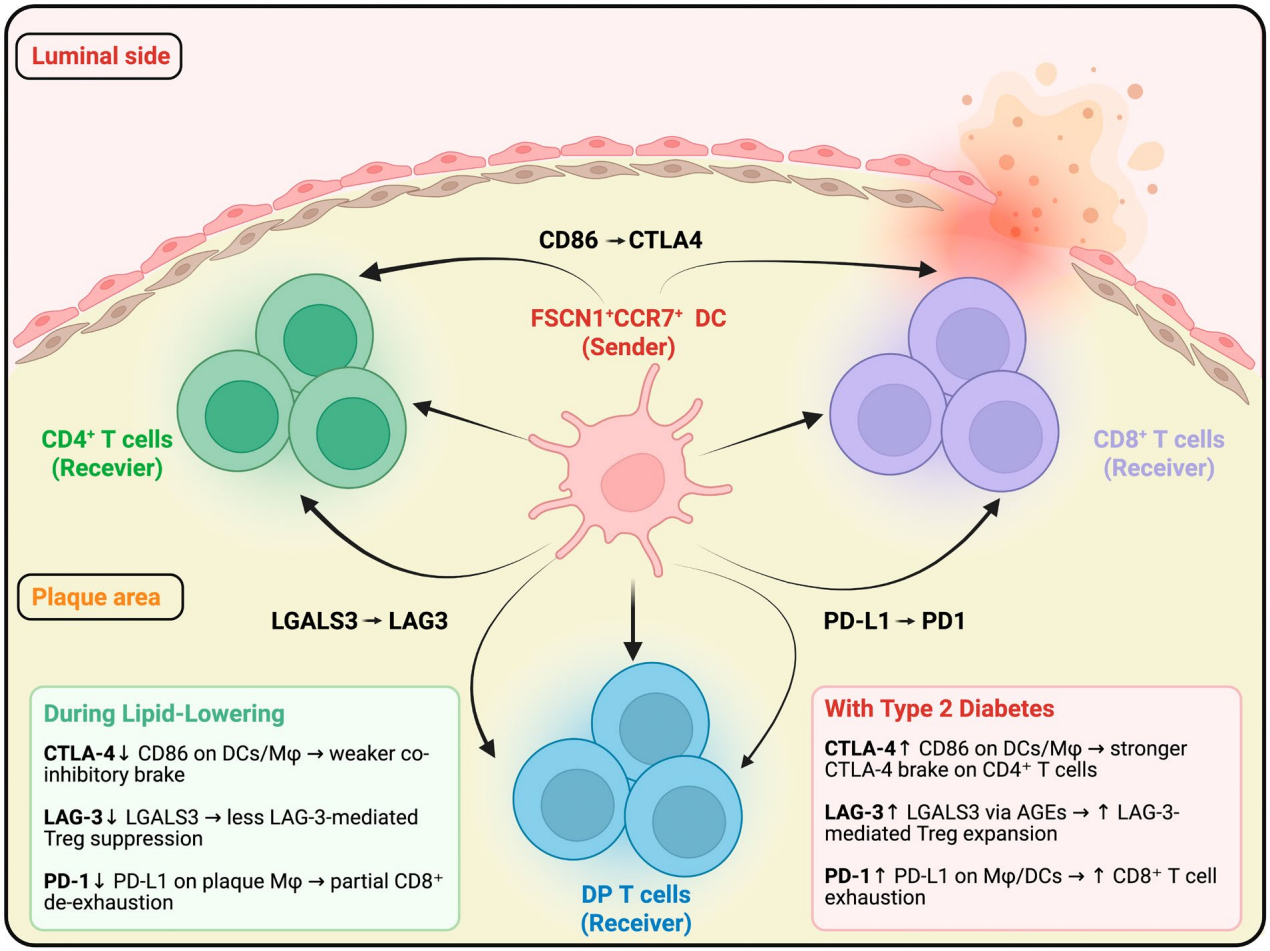
Schematic overview of immune checkpoint interactions within the human atherosclerotic plaque microenvironment. FSCN1^+^CCR7^+^ dendritic cells (DCs) interact with multiple T‐cell subsets through canonical inhibitory checkpoint pathways, including CD86–CTLA‐4, LGALS3–LAG3, and PD‐L1–PD‐1 signaling. These interactions modulate T‐cell activation, exhaustion, and regulatory function within plaques, influencing local inflammation and immune homeostasis. CD4^+^ T cells, CD8^+^ T cells, and double‐positive (DP) T cells are key cellular targets of checkpoint‐mediated regulation. Metabolic context alters this checkpoint landscape. During lipid lowering, reduced expression of CD86, LGALS3, and PD‐L1 is associated with weaker inhibitory signaling, diminished regulatory T‐cell suppression, and partial reversal of CD8^+^ T‐cell exhaustion. In contrast, type 2 diabetes enhances checkpoint signaling through increased CD86 expression, AGE‐driven LGALS3 induction, and elevated PD‐L1 expression on macrophages and DCs.

The atherosclerotic plaque is an organized immune microenvironment governed by the same checkpoint axes targeted by ICI therapy in tumors. Comprehensive immune profiling of human carotid and coronary atherosclerotic plaques by single‐cell RNA sequencing, multiplexed immunofluorescence, and spatial protein validation demonstrated that plaques harbor a multilineage, functionally active checkpoint network (Figure 2) [113]. This network spans PD‐1, PD‐L1, CTLA4, LAG3, TIM3, TIGIT, and additional investigational checkpoint pathways across resident T cells, macrophages, and dendritic cells. A central finding was the identification of mature regulatory CCR7^+^FSCN1^+^ dendritic cells as a plaque checkpoint hub, analogous to dendritic cell states described in tumors, coordinating PD‐1‐, CTLA4‐, LAG3‐, TIM3/galectin‐9‐ and TIGIT‐related interactions with effector and regulatory T‐cell populations. This architecture provides a direct cellular substrate through which systemic checkpoint blockade may unintentionally disrupt immune restraint within atherosclerotic lesions.

Several features of this landscape carry direct mechanistic implications for ICI‐associated vascular toxicity. PD‐1‐, LAG3‐ and TIM3‐expressing effector/memory CD8^+^ T cell populations were embedded within checkpoint ligand‐rich myeloid niches, while PD‐L1, CD86, galectin‐3, and galectin‐9 were expressed by dendritic‐cell and macrophage subsets capable of engaging T cell inhibitory receptors. Importantly, functional validation in human carotid plaque explants showed that PD‐1 blockade increased activation, inflammatory, and cytotoxic features of plaque T cells, including IFN‐γ induction in CD8^+^ T cells and granzyme B induction in CD4^+^ T cells. Thus, plaque checkpoint expression is not merely a static marker of immune exhaustion, but represents an active regulatory circuit that can be pharmacologically perturbed by ICI therapy.

The study further showed that cardiometabolic context modifies this checkpoint architecture. Type 2 diabetes altered PD‐1‐ and CTLA4‐related immune‐cell communication, while lipid lowering reshaped ICI‐relevant ligand‐receptor interactions, including PD‐L1–PD‐1, CD86–CTLA4, and galectin‐3–LAG3 axes. These findings suggest that vascular risk factors and their treatment influence the degree and configuration of checkpoint‐mediated immune restraint within plaques before cancer therapy is initiated. This cardiometabolic stratification of the plaque checkpoint landscape may help explain why patients with established atherosclerosis or residual inflammatory risk appear particularly vulnerable to ICI‐associated myocardial infarction and stroke.

### 4.2 ICI Therapy Accelerates Atherosclerosis: Preclinical and Clinical Evidence

The mechanistic basis of ICI cardiovascular toxicity was first defined through histopathological characterization of fulminant myocarditis after combined nivolumab and ipilimumab therapy. These cases showed dense CD8^+^ T cell and macrophage infiltration of the myocardium and suggested clonal overlap between tumor‐ and heart‐infiltrating T cells [10]. This established ICI myocarditis as an immune‐mediated loss‐of‐tolerance syndrome rather than a nonspecific chemotherapy‐like cardiotoxicity. Genetic mouse models subsequently showed that disruption of PD‐1–PD‐L1 signaling can unleash autoreactive T‐cell responses against cardiac tissue, consistent with the role of myocardial PD‐L1 and PD‐L2 expression in restraining immune surveillance of the heart [187, 188]. Further molecular studies identified converging mechanisms, including loss of CTLA‐4‐mediated tolerance, reinvigoration of cardiac antigen‐reactive memory T cells by PD‐1 blockade, and IFN‐γ‐driven upregulation of MHC class II on cardiomyocytes, which may sustain local antigen presentation within the myocardium [189]. Although myocarditis remains the prototypical fulminant cardiac irAE, the same core principle, removal of inhibitory checkpoints that restrain tissue T cell activation, also provides a rationale for vascular inflammation and plaque progression.

The convergence of genetic, pharmacological, and meta‐analytic preclinical evidence establishes checkpoint inhibition as a pro‐atherogenic intervention across multiple mouse models and checkpoint targets. The foundational observations came from genetic loss‐of‐function studies targeting the PD‐1 axis. In LDLR‐deficient mice, combined deletion of PD‐L1 and PD‐L2 produced significantly larger atherosclerotic lesions throughout the aorta, with a marked increase in lesional CD4^+^ and CD8^+^ T cells and elevated systemic TNF‐α, demonstrating that the PD‐1 ligand pathway constitutively restrains pro‐atherogenic T cell activation [190]. This was extended by Bu et al., who showed that PD‐1‐deficient LDLR‐knockout mice developed larger lesions with greater T cell and macrophage infiltration and enhanced CD8^+^ cytotoxic T cell activity; pharmacological anti‐PD‐1 antibody blockade reproduced this phenotype in hypercholesterolemic mice, establishing that therapeutic blockade accelerates lesion inflammation [191]. The reciprocal experiment confirmed the principle: transgenic overexpression of CTLA‐4 in ApoE‐deficient mice reduced atherosclerotic lesion formation and suppressed intraplaque macrophage and CD4^+^ T cell accumulation, while simultaneously dampening systemic dendritic cell co‐stimulatory capacity [192]. Direct modeling of therapeutic ICI in atherosclerosis‐prone mice, demonstrated that antibody‐mediated CTLA‐4 inhibition produced a two‐fold increase in aortic arch plaque area, drove a T cell‐dominant inflammatory phenotype with reduced smooth muscle cell and collagen content, and upregulated endothelial ICAM‐1, all hallmarks of plaque vulnerability [193]. Combined anti‐CTLA‐4 and anti‐PD‐1 treatment induced qualitative rather than quantitative plaque progression, generating a 2.7‐fold expansion of intraplaque CD8^+^ cytotoxic T cells and a 3.9‐fold increase in necrotic core area alongside endothelial adhesion molecule upregulation, while leaving myeloid‐driven vascular inflammation unaffected, a mechanistically important dissociation indicating that dual ICI remodels plaque composition through the adaptive immune compartment specifically [194]. The class effect extends to emerging checkpoint targets: anti‐LAG3 monotherapy in hypercholesterolemic mice doubled intraplaque T cell density, and combined anti‐LAG3 plus anti‐PD‐1 had additive effects on T‐cell activation and plaque infiltration, foreshadowing the cardiovascular risk profile of novel LAG‐3‐targeting regimens entering clinical practice [195]. These individual observations are quantitatively synthesized by a systematic review and meta‐analysis of 14 preclinical studies, which found that checkpoint inhibition consistently increased atherosclerotic plaque size by 53% across PD‐1, PD‐L1, CTLA‐4, and LAG‐3 targets (RoM 1.53) with plaque inflammation dominated by greater CD4^+^, CD8^+^, and macrophage content; conversely, checkpoint stimulation reduced plaque burden by 28% (RoM 0.72), confirming a bidirectional, causally coherent relationship between checkpoint tone and atherogenesis [196].

Clinical characterization studies have defined ICI myocarditis as an early‐onset, high‐mortality inflammatory syndrome with frequent arrhythmias, conduction disease, troponin elevation, and variable left ventricular dysfunction. In a prospective case series of 35 patients, arrhythmia and cardiac dysfunction emerged as dominant clinical manifestations, and high‐sensitivity troponin was the most sensitive early biomarker [197]. A diagnostic framework subsequently integrated cardiac MRI with T2‐weighted oedema imaging and late gadolinium enhancement as reference tools for myocarditis confirmation, alongside treatment algorithms including high‐dose corticosteroids, abatacept, and ruxolitinib for steroid‐refractory cases [198]. Longitudinal cardiac imaging in ICI‐treated patients showed that left ventricular ejection fraction trajectory, early troponin kinetics, and cardiac MRI edema extent predict myocardial dysfunction severity and probability of long‐term recovery [199]. However, myocarditis represents only one manifestation of ICI‐associated cardiovascular inflammation. Vascular toxicity requires distinct endpoints, biomarkers, and surveillance strategies.

Atherosclerosis acceleration by ICI therapy has been documented across clinical imaging platforms. In a matched cohort study, cancer patients receiving ICIs had higher rates of major adverse cardiovascular events and accelerated atherosclerotic plaque progression on computed tomography imaging compared with matched cancer controls [200]. Serial CTA in lung cancer patients subsequently confirmed progression of non‐calcified coronary plaque volume over 12 months on ICI therapy, with plaque progression correlating with systemic inflammatory biomarker elevation, supporting IFN‐γ‐driven vascular inflammation as a plausible pathway [201]. In melanoma patients receiving ICI, serial computed tomographic assessment of five aortic segments demonstrated significant plaque thickness progression across all sites, ranging from 3.0% to 8.0% per year, with 75% of patients experiencing substantial plaque growth in at least one segment [202]. Notably, the number of plaques remained stable in the majority of arterial segments, suggesting that ICI therapy predominantly accelerates existing plaque rather than driving de novo lesion formation. ICI combination therapy showed a trend toward greater plaque progression risk compared with monotherapy (OR 2.10), while antihypertensive drug use was associated with a significant reduction in risk (OR 0.48), raising the possibility that cardiovascular risk factor optimization may partially mitigate ICI‐accelerated plaque growth. In a parallel imaging cohort of women with cancer treated with ICI, 5% developed an atherosclerotic cardiovascular event, prior cardiovascular disease was the strongest predictor of post‐ICI ASCVD (HR 2.71), and serial imaging confirmed annual non‐calcified plaque progression rates of 7%, consistent with the rates observed in mixed‐sex cohorts [203, 204]. Serial ^18^F‐fluorodeoxyglucose positron emission tomography (^18^F‐FDG‐PET) has provided complementary evidence for ICI‐associated arterial inflammation. In a prospective cohort of 156 cancer patients undergoing serial FDG‐PET, ICI‐treated patients showed that arterial FDG uptake increases by 2.5% per year versus 0.8% per year in ICI‐naïve controls, with consistent effects across aortic and carotid landmarks [205]. The authors noted that the absolute FDG signal change was modest relative to individual variability, and that FDG uptake reflects macrophage‐driven metabolic activity rather than the lymphocyte‐mediated plaque remodeling captured by CTA. These modalities are therefore complementary: CTA characterizes plaque burden, volume, and composition, while FDG vascular uptake provides an indirect marker of vascular metabolic inflammation. At the population level, a meta‐analysis of 26 cohort studies involving over 109,000 cancer patients confirmed significantly higher risks of all‐grade major adverse cardiovascular events and pericardial effusion in ICI‐treated patients compared with non‐ICI controls [206]. ICI treatment, older age, male sex, and prior radiotherapy were each independently associated with MACE risk, identifying a clinically actionable risk profile for cardiovascular surveillance in ICI‐treated populations. Accelerated plaque disease and ICI myocarditis likely share upstream immune‐effector mechanisms, particularly reinvigoration of antigen‐specific T cells by checkpoint blockade, but diverge in tissue pathology, clinical presentation, imaging signatures, and biomarker kinetics [207]. The available evidence therefore supports ICI‐associated atherosclerosis acceleration as an emerging vascular toxicity of checkpoint blockade that operates in parallel with direct myocardial inflammation, through overlapping immune pathways but distinct vascular mechanisms. Despite converging mechanistic, human plaque, and imaging evidence, ICI‐accelerated atherosclerosis remains clinically underdefined. The existing evidence base relies mainly on matched retrospective cohort studies [200, 201], single‐center serial imaging series with heterogeneous endpoint definitions [202, 205], and population‐level pharmacovigilance data not designed to detect subclinical vascular progression. To date, no major randomized ICI trial has incorporated pre‐specified coronary plaque progression, serial arterial inflammation quantification, or adjudicated vascular MACE as a primary cardiovascular endpoint in ICI‐treated cancer populations with systematic cardiovascular surveillance. This evidence gap has direct clinical consequences. Optimal surveillance intervals, intervention thresholds, and ICI‐specific preventive strategies remain uncertain. Although statins, colchicine, and PCSK9 inhibitors have strong rationale in broader ASCVD, their efficacy in preventing ICI‐associated plaque inflammation and vascular events remains untested in dedicated prospective cardio‐oncology trials. The field now has the mechanistic rationale and imaging tools required to move from observational association to prospective trials testing whether vascular inflammation can be monitored, modified, and prevented during ICI therapy.

## 5 Reverse Cardio‐Oncology: How Cardiovascular Disease Promotes Cancer

### 5.1 Epidemiological Evidence for Reverse Cardio‐Oncology

**FIGURE 3:**
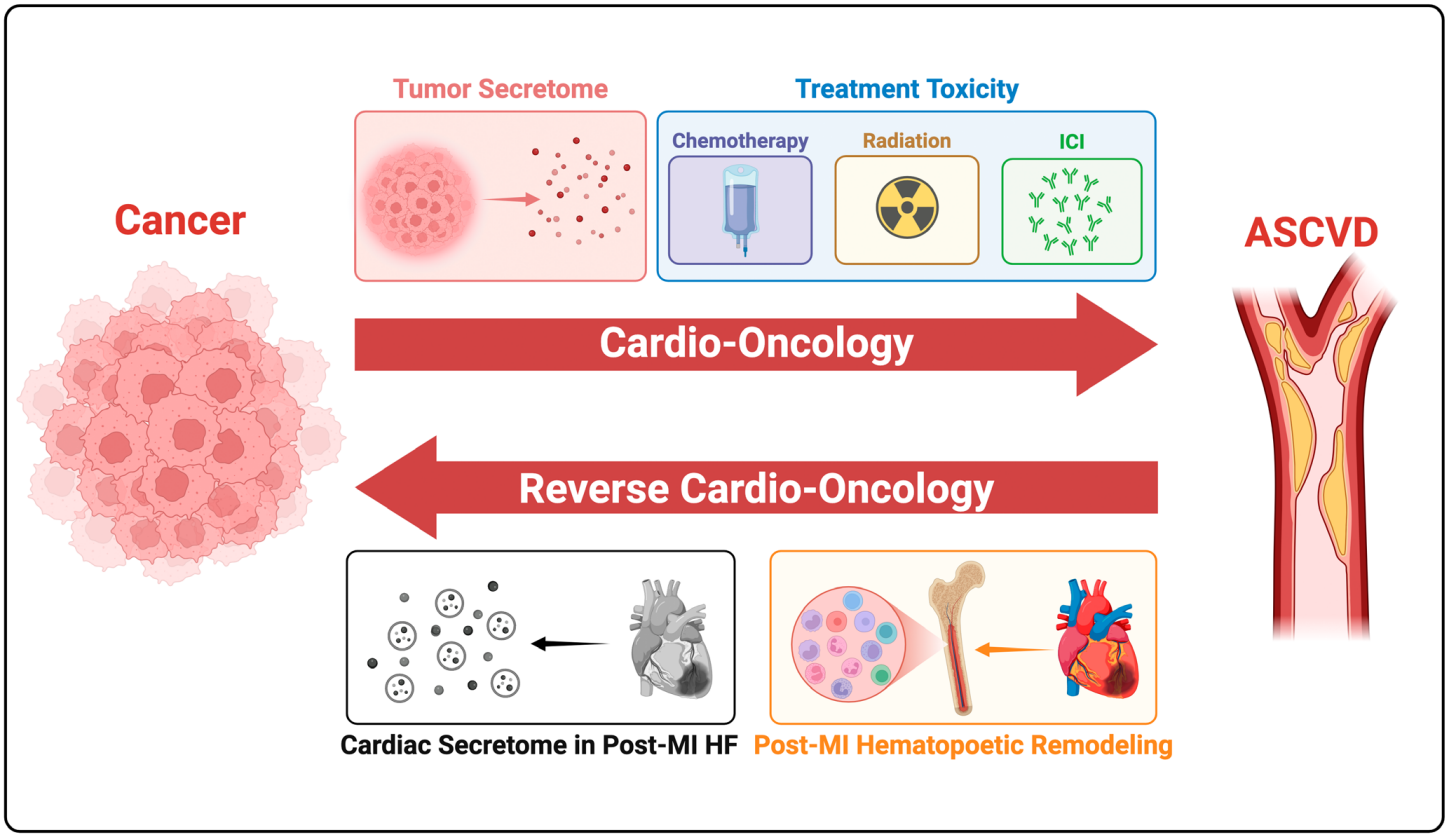
Schematic illustration of the reciprocal relationship between cancer and atherosclerotic cardiovascular disease (ASCVD). In the traditional cardio‐oncology paradigm, tumors promote cardiovascular pathology through the release of circulating tumor‐derived factors (tumor secretome) and through cardiovascular toxicities associated with cancer therapies, including chemotherapy, radiation, and immune checkpoint inhibitors. These processes contribute to vascular inflammation, endothelial dysfunction, and accelerated atherosclerosis. Conversely, in the emerging field of reverse cardio‐oncology, cardiovascular disease influences cancer progression. Myocardial infarction (MI) and heart failure (HF) induce systemic alterations including hematopoietic remodeling in the bone marrow and release of cardiac‐derived secreted factors that reshape immune responses and the tumor microenvironment, thereby promoting tumor growth and metastasis. Together, these bidirectional interactions highlight the complex crosstalk between cardiovascular disease and cancer and underscore the need for integrated mechanistic and therapeutic approaches across both disciplines.

The epidemiological relationship between ASCVD and cancer is not one of passive co‐occurrence, but of bidirectional association and biological reinforcement (Figure 3). Among more than 3.2 million US cancer patients, cardiovascular disease was the leading non‐cancer cause of death, with standardized mortality ratios for cardiac death exceeding 2.0 across multiple malignancy subtypes after multivariable adjustment [208]. Cancer patients and survivors harbor significantly higher atherosclerotic plaque burden independent of traditional cardiovascular risk factors, as demonstrated by invasive coronary angiography showing both greater plaque extent and more complex plaque morphology in patients with cancer compared to age‐matched controls [209]. Beyond vascular changes, a new cancer diagnosis was independently associated with significantly increased risks of cardiovascular death, stroke, heart failure, and pulmonary embolism across multiple tumor types in a prospective population‐based cohort, with cardiovascular risk highest among genitourinary, gastrointestinal, thoracic, nervous system, and hematologic malignancies [210]. Evidence for the reverse direction has also strengthened. In more than 27 million cancer‐free individuals, patients with ASCVD carried a 20% higher hazard of developing cancer, with subtype analyses revealing specific associations with lung, bladder, liver, colon, and hematologic malignancies [211]. In a cohort of 32,095 consecutive patients with acquired CVD enrolled in the Sakakibara Health Integrative Profile study, those with atherosclerotic CVD, including coronary artery, aortic, and peripheral artery diseases, had more than twice the cancer incidence of patients with non‐atherosclerotic CVD, reinforcing that atherosclerosis may constitute an independent cancer risk factor [212]. Peripheral artery disease (PAD) further independently elevated subsequent cancer risk in the Atherosclerosis Risk in Communities (ARIC) cohort of over 13,000 adults followed for a median of 25 years, with the association being particularly pronounced for lung cancer among ever smokers [213]. An elevated risk of hematologic malignancies following acute MI (AMI) has been documented in a large retrospective cohort study matching 103,686 patients with AMI with individuals with no history of AMI or hematologic malignancies [214]. Post‐MI heart failure carries greater cancer risk than MI without heart failure, suggesting a dose‐dependent relationship between the severity of cardiac dysfunction and its pro‐oncogenic influence [215]. Importantly, the relationship extends beyond cancer incidence to cancer prognosis: in a clinical cohort of early‐stage breast cancer patients, Koelwyn et al. [164] demonstrated that cardiovascular events occurring after cancer diagnosis were independently associated with significantly increased risks of both recurrence (+59%) and cancer‐specific death (+60%) in a cohort of 1724 early stage breast cancer patients. Corroborating these findings, a large cohort study encompassing over 30,000 breast cancer survivors showed that incident MI and/or heart failure was associated with a nearly four‐fold higher hazard of cancer‐specific death [216]. While the forward cardio‐oncology axis, whereby tumor‐derived inflammatory mediators and cancer therapies including chemotherapies, thoracic radiation, and immune checkpoint inhibitors promote atherosclerosis and myocardial injury, has been extensively characterized [7, 92, 217], the mechanisms underlying this reverse relationship have only recently begun to be defined.

### 5.2 Post‐MI Hematopoietic Remodeling and Altered Antitumoral Immunity

Myocardial infarction does not only injure the heart but also reprograms the bone marrow in ways that durably reshape systemic immunity and accelerate tumor progression. In a landmark contribution to the field of reverse cardio‐oncology, Koelwyn et al. [164] demonstrated that MI accelerates breast cancer outgrowth and cancer‐specific mortality in both the E0771 syngeneic and MMTV‐PyMT genetically engineered mouse models by enriching the tumor microenvironment with monocytic myeloid‐derived suppressor cells and regulatory T cells. The group showed that MI epigenetically reprograms bone marrow Ly6C^hi^ monocytes toward an immunosuppressive phenotype—sustained in both the circulation and the tumor—where they actively inhibit anti‐tumor T cell activity; critically, depletion of these cells slowed tumor development, establishing causality. ATAC‐seq analyses revealed persistent chromatin remodeling in bone marrow monocyte progenitors, indicative of durable epigenetic imprinting of the myeloid compartment. Notably, bone marrow transplantation experiments confirmed that these epigenetic alterations are sufficient to accelerate tumor growth in naïve recipient mice, establishing that MI induces a systemic, long‐lasting pro‐tumorigenic reprogramming of hematopoietic reservoirs.

Whether this form of hematopoietic reprogramming is specific to coronary ischemia or reflects a broader response to tissue injury is addressed by studies of peripheral ischemia [165]. Hind limb ischemia similarly accelerates tumor growth and induces a sustained shift toward myeloid‐biased hematopoiesis at the expense of lymphoid output. Single‐cell and epigenomic analyses identify a progenitor state characterized by activation of inflammatory pathways, including NLRP3‐dependent signaling, and enrichment of aging‐associated transcriptional programs. As with MI, bone marrow transplantation after hind limb ischemia in tumor bearing mice confirmed that this state is heritable across hematopoietic reconstitution. Together, these observations define a paradigm in which ischemic injury, whether myocardial or peripheral, induces a durable epigenetic imprint within hematopoietic stem and progenitor cells, skewing myeloid output toward an immunosuppressive phenotype that creates a systemic environment permissive to tumor progression. This provides a compelling mechanistic framework for the epidemiologically observed association between ASCVD and increased cancer risk in humans.

### 5.3 The Cardiac Secretome: Extracellular Vesicles and Neurotrophic Signals

The failing heart functions as an active endocrine organ whose post‐ischemic secretome exerts systemic pro‐tumorigenic effects through multiple mechanistically distinct pathways, implicating the myocardium as a remote driver of cancer progression independent of immune cell remodeling [6, 164, 218]. Foundational evidence for this paradigm comes from Meijers et al. [219] who demonstrated for the first time that the failing heart directly stimulates tumor growth through circulating secreted factors. Using MI‐induced heart failure in tumor‐prone APC^min^ mice, and confirming findings in a heterotopic transplantation model that explicitly ruled out hemodynamic confounding, they observed a 2.4‐fold increase in intestinal tumor load, with SerpinA3 emerging as a potent secreted candidate, driving colon cancer cell proliferation via Akt‐S6 phosphorylation. Human validation in the PREVEND cohort (*n* = 8319) further showed that elevated cardiac biomarkers independently predicted new‐onset cancer over 11.5 years, establishing heart failure as an independent risk factor for incident cancer. A novel heart‐to‐tumor signaling axis has been identified in which post‐MI cardiomyocytes release exosomes enriched in miR‐22‐3p, a microRNA significantly upregulated in plasma exosomes of human heart failure patients [220]. In LLC lung carcinoma and K7M2 osteosarcoma xenograft models, post‐MI plasma exosomes suppressed ferroptosis sensitivity in tumor cells by downregulating *ACSL4*, a key regulator of ferroptosis‐associated lipid peroxidation [220, 221]. This attenuated the antitumor efficacy of the canonical ferroptosis inducer erastin and restored cancer cell proliferation, invasion, and migration. Cardiac‐targeted inhibition of miR‐22‐3p significantly abrogated these pro‐tumorigenic effects, establishing miR‐22‐3p as a causal mediator of cardiomyocyte‐to‐tumor signaling and a potential cardiac‐specific therapeutic target [220].

Cardiac mesenchymal stromal cells (cMSCs) from post‐MI failing hearts have been identified as a quantitatively and qualitatively distinct source of pro‐tumorigenic small extracellular vesicles (sEVs) [222]. Post‐MI cMSCs produced twice as many sEVs as controls, with proteomic cargo enriched in osteopontin, periostin, IL‐6, galectin‐3, TNF‐α, and VEGF, alongside tumor‐promoting miRNAs including miR‐221 and miR‐21. In vitro, cMSC‐sEVs accelerated proliferation and migration of lung and colon cancer cells and activated macrophages toward a proangiogenic, immunosuppressive phenotype marked by PD‐L1 upregulation. Causality was established in vivo by adoptive transfer of post‐MI cMSC‐sEVs, which reproduced accelerated tumor growth in mice without left ventricular dysfunction, demonstrating a cell‐source‐specific mechanism. Spironolactone reduced cMSC‐sEV secretion by 28% and suppressed tumor growth in post‐MI mice without direct antitumor activity, providing proof of concept that reducing cardiac vesicle burden may represent a clinically translatable intervention strategy [218, 222].

Beyond vesicle‐mediated signaling, the ischemic myocardium co‐opts neurotrophic pathways to remotely accelerate tumor growth. MI drives sustained elevation of circulating nerve growth factor (NGF) in a syngeneic 4T1 mammary tumor model, where circulating NGF activates TrkA receptors on tumor cells and triggers downstream PI3K‐AKT signaling; RNA sequencing confirmed enrichment of cell cycle and proliferative gene sets in tumor tissue [223]. TrkA inhibition by siRNA knockdown and the small‐molecule inhibitor GW441756 abolished NGF‐driven proliferative signaling in vitro and suppressed tumor volume and Ki67‐positive cell fraction in vivo, without affecting left ventricular function or infarct size, positioning TrkA as a discrete and cardiac‐safe therapeutic target at the MI‐tumor interface [223].

Collectively, these findings reframe the post‐ischemic heart as a cellularly compartmentalized secretory organ, with cardiomyocytes, cardiac mesenchymal stromal cells, and the myocardium as a neurohumoral source each contributing mechanistically distinct effector signals: exosomal miRNAs suppressing ferroptosis, cytokine‐ and miRNA‐laden sEVs remodeling the tumor immune microenvironment, and circulating NGF activating tumor cell proliferative programs. These orthogonal oncogenic pathways represent independent therapeutic targets in the cardio‐oncology setting.

## 6 Clinical and Therapeutic Implications

### 6.1 Dual‐Action Anti‐Inflammatory Strategies: Canakinumab

The IL‐1β axis is the most clinically validated dual‐action anti‐inflammatory target at the intersection of ASCVD and cancer. IL‐1β hypersecretion by TET2‐mutant macrophages drives accelerated atherosclerosis, and IL‐1β blockade abrogates this phenotype, providing the preclinical mechanistic foundation for clinical IL‐1β targeting [64]. In the CANTOS trial, a randomized, placebo‐controlled trial of the anti‐IL‐1β monoclonal antibody canakinumab in 10,061 post‐MI patients with elevated hsCRP, a 15% relative risk reduction in recurrent major adverse cardiovascular events was demonstrated independently of lipid lowering [224]. A pre‐specified secondary analysis of CANTOS demonstrated a significant reduction in incident cancer mortality (HR 0.49) and cancer incidence, with the strongest effect for lung cancer, establishing clinical proof of concept for the dual cardiovascular‐oncologic benefit of IL‐1β inhibition [225]. Subsequent trials of canakinumab in combination with immunotherapy (CANOPY‐1 and CANOPY‐2) in non‐small cell lung cancer did not prolong disease‐free survival, and the lessons this experience holds for IL‐1β inhibition as an oncologic repositioning strategy have been reviewed [226, 227, 228]. Exploratory analyses of CANTOS lung cancer participants suggest that IL‐1β inhibition may have differential activity across non‐small cell lung cancer molecular subtypes, including those with distinct oncogenic driver mutations [229], a hypothesis requiring prospective evaluation. TET2 mutation carriers who received canakinumab in the CANTOS trial showed greater reductions in cancer incidence than non‐TET2 carriers, suggesting that TET2 genotype may be a predictive biomarker for IL‐1β inhibition benefit in cancer prevention [230]. Canakinumab also reduced incident anemia in CHIP carriers and reversed proteomic signatures of myeloid activation in CANTOS participants, suggesting that IL‐1β inhibition may normalize CHIP‐associated inflammatory signaling beyond reducing cardiovascular events [231].

### 6.2 Dual‐Action Anti‐Inflammatory Strategies: Colchicine

Colchicine, a microtubule‐disrupting agent with broad anti‐inflammatory properties including neutrophil degranulation inhibition, NETosis suppression, and NLRP3 inflammasome disruption, has demonstrated cardiovascular efficacy in two landmark randomized trials. Low‐dose colchicine (0.5 mg daily) initiated within 30 days of MI significantly reduced major adverse cardiovascular events (HR 0.77, 95% CI 0.61–0.96) in the COLCOT trial, with the greatest absolute benefit for stroke and coronary revascularization [232]. These benefits extended to patients with stable chronic coronary disease in the LoDoCo2 trial [233], where colchicine significantly reduced cardiovascular events. More recently, direct evidence for plaque‐stabilizing effects of colchicine was shown in patients with acute coronary syndrome: colchicine increased minimal fibrous cap thickness (87 vs. 52 μm; difference 34.2 μm) and reduced lipid arc (−10.5) [234]. Colchicine 0.5 mg daily did not reduce the primary composite of cardiovascular death, recurrent MI, stroke, or unplanned revascularization compared with placebo in the CLEAR SYNERGY trial (HR 0.99) despite significant CRP reduction at 3 months, representing a neutral result in the immediate post‐MI/post‐PCI setting [235]. In addition, colchicine prevents CHIP‐driven accelerated atherosclerosis in TET2‐mutant murine models through NLRP3 inflammasome‐disruption [65], positioning it as a compelling dual‐action candidate for patients with CHIP who simultaneously carry elevated cardiovascular and cancer risk. Colchicine's anti‐NETosis effects provide an additional mechanistic rationale for its dual cardiovascular‐oncologic protective potential, given the recognized role of NETs in both vascular thrombosis and cancer metastatic seeding [12]. Finally, colchicine's tubulin‐binding mechanism adds a further dimension of oncologic interest: it binds the colchicine site at the intradimer interface of the tubulin heterodimer, preventing the conformational change required for microtubule assembly [18], the same pharmacological target exploited by vascular disrupting agents in oncology drug development, including combretastatins and ombrabulin [236]. Whether the concentrations achieved with cardiovascular low‐dose regimens are sufficient for direct anti‐mitotic activity in cancer cells remains untested, but meta‐analysis of colchicine cardiovascular trials found no increase in newly diagnosed cancers with colchicine compared with placebo [237], providing a safety baseline from which cancer endpoint trials could be designed.

### 6.3 Statin Pleiotropic Effects and Immune Modulation

Statins exert pleiotropic anti‐inflammatory effects through mevalonate pathway inhibition beyond LDL lowering, including downregulation of ICAM‐1 and VCAM‐1, expansion of regulatory T cells, inhibition of Rho GTPase‐mediated macrophage activation, and reduction of circulating IL‐6 and hsCRP [238]. Among statin‐treated patients, residual inflammation measured by hsCRP is a stronger determinant of cardiovascular death and all‐cause mortality than on‐treatment LDL‐C, reinforcing the notion that the inflammatory component of cardiovascular risk is independently targetable and that statin benefit operates partly through immune modulation [239, 240]. Observational data associating statin use with reduced cancer incidence have been reported for several malignancy types, with the most consistent evidence in colorectal cancer among patients with inflammatory bowel disease and in hepatocellular carcinoma. However, randomized confirmatory trials with prespecified cancer endpoints are still lacking [241]. In the cardio‐oncology setting specifically, concomitant statin use has not been shown to significantly improve overall survival in ICI‐treated patients in available retrospective cohorts [242, 243], underscoring that the immune modulatory potential of statins at the tumor‐cardiovascular interface warrants prospective evaluation.

## 7 Conclusion and Future Directions

The relationship between cardiovascular disease and cancer increasingly reflects more than epidemiological coincidence. Single‐cell technologies, epigenomic profiling, and large‐scale genomic cohorts have reframed these conditions as biologically intertwined disease processes linked by shared inflammatory, immunological, and hematopoietic mechanisms. Clonal hematopoiesis, particularly through TET2‐ and DNMT3A‐mutant myeloid expansion, can amplify vascular inflammation while shaping immunosuppressive tumor niches, positioning CHIP as a molecular fulcrum between two leading causes of mortality in the developed world. Similarly, the identification of functional immune checkpoint networks within human atherosclerotic plaques provides a mechanistic basis for ICI‐associated vascular toxicity and a framework for precision vascular risk stratification before cancer immunotherapy. In parallel, experimental myocardial infarction can induce durable hematopoietic and secretome‐mediated reprogramming that promotes primary tumor growth and metastasis. Together, these pathways define a therapeutically accessible communication axis linking the injured heart, the immune system, and the tumor microenvironment.

The most pressing unanswered questions are catalogued in Tables 1 and 2. Whether the CHIP variant allele frequency threshold that predicts cardiovascular risk is the same threshold relevant to cancer susceptibility remains undefined. Similarly, connecting checkpoint biology observed in human carotid plaques to individual vascular event risk before immune checkpoint inhibitor therapy remains an unmet clinical need. Agents with plausible dual cardiovascular and oncologic relevance, including NLRP3 inhibitors, IL‐6 receptor blockade, and colchicine, have generally been tested in trials powered for one disease domain rather than both simultaneously. CANTOS revealed a striking reduction in lung cancer incidence as a secondary finding of a cardiovascular outcomes trial, but this signal has not yet been followed by a purpose‐designed trial in CHIP carriers with co‐primary cardiovascular and cancer‐related endpoints.

**TABLE 1:** Dual‐action anti‐inflammatory strategies at the ASCVD–cancer intersection.

Agent	Primary mechanism	Key ASCVD evidence	Cancer signal	Key gaps
Canakinumab (anti‐IL‐1β mAb)	Selective IL‐1β blockade; NLRP3 effector suppression	CANTOS (*n* = 10,061 post‐MI): HR 0.85 for MACE, LDL‐independent [224]	Cancer mortality HR 0.49; lung cancer incidence reduced [225]	No RCT with cancer as primary endpoint No CHIP‐stratified cancer prevention trial Cost and infection risk limit broad use
Colchicine	NLRP3 inflammasome suppression; neutrophil degranulation + NETosis inhibition	COLCOT: HR 0.77 post‐MI [232]; LoDoCo2: stable CAD; plaque stabilization on OCT	No cancer endpoint in any RCT; anti‐NETosis mechanism plausible	Cancer prevention rationale entirely preclinical No human CHIP‐stratified trial data NETosis–cancer link clinically untested
Statins/PCSK9 inhibitors	LDL lowering + pleiotropic anti‐inflammatory effects (IL‐6/CRP reduction, Treg expansion, Rho GTPase inhibition)	25%–35% relative MACE reduction (4S, JUPITER, HPS); dual low LDL + low hsCRP superior to either alone	Observational cancer‐mortality reduction; no RCT cancer endpoint; cholesterol shapes T cell cytotoxic function	No RCT with cancer as primary endpoint Observational cancer data subject to confounding CHIP‐stratified benefit undefined
Emerging: NLRP3 inhibitors; IL‐6 blockade	Upstream IL‐1β/IL‐18 block (NLRP3i); STAT3 suppression + myeloid reprogramming (IL‐6R mAb)	IL‐6 blockade reduces CHIP‐driven atherosclerosis in mice [66]; NLRP3i: preclinical only	Early oncology trials only; no dual CVD–cancer endpoint data for any agent	No cardiovascular RCT for either agent All dual‐benefit evidence preclinical CHIP genotype‐response unmapped in humans

Abbreviations: CAD, coronary artery disease; CHIP, clonal hematopoiesis of indeterminate potential; hsCRP, high‐sensitivity C‐reactive protein; mAb, monoclonal antibody; MACE, major adverse cardiovascular events; RCT, randomized controlled trial.

**TABLE 2:** Knowledge gaps and open questions across the ASCVD–cancer interface.

Theme	Current evidence	Key open questions
CHIP as a dual‐disease biomarker	2‐fold elevated ASCVD risk [4, 5] IL‐1β/IL‐6 effector mechanism defined in murine models [64, 66] Established pre‐malignant state conferring cancer risk [3]	No validated CHIP‐specific cancer surveillance protocol No prospective RCT with cancer as primary endpoint in CHIP carriers Optimal VAF threshold for risk stratification undefined
Post‐MI hematopoietic remodeling	HSPC epigenetic reprogramming → myeloid‐biased immunosuppression → tumor acceleration [164] CCR2 depletion abrogated 56% of MI‐accelerated tumor growth Peripheral ischemia installs same HSPC program via NLRP3–IL‐1β axis [165]	Human post‐MI HSPC epigenetic data lacking Optimal intervention window undefined No clinical trial targeting post‐MI hematopoietic remodeling
Atherosclerotic plaque checkpoint biology	PD‐1/CTLA‐4/LAG‐3 network spatially organized in human carotid plaques [113] CCR7 + FSCN1+ DC identified as central immunoregulatory hub Anti‐PD‐1 activates plaque T cells ex vivo; T2D and lipid lowering remodel checkpoint interactions	Plaque checkpoint burden not linked to individual ICI vascular event risk Checkpoint restoration as a plaque‐stabilizing strategy clinically untested No validated peripheral biomarker surrogate for plaque immunophenotype
ICI‐accelerated atherosclerosis	CT angiography confirms non‐calcified plaque progression in ICI‐treated patients [200, 201] Baseline CAC score predicts vascular events pre‐ICI [203] Acceleration confirmed across multiple checkpoint targets in preclinical models	No RCT of cardioprotective co‐intervention during ICI therapy No validated pre‐ICI biomarker for individual vascular risk Mechanism how ICI aggravate atherosclerosis unknown
Cardiac secretome and cancer promotion	Post‐MI sEVs (miR‐21/miR‐146a) reprogram TME macrophages and enhance tumor ferroptosis resistance [222] NGF/TrkA axis links ischemic myocardium to mammary tumor proliferative signaling [223]	No human circulating sEV cancer biomarker validated prospectively Pharmacological interruption of cardiac–tumor EV communication not clinically tested Relevance beyond breast cancer models unknown
Therapeutic translation	Canakinumab: MACE reduction + lung cancer benefit in CANTOS [224, 225] Colchicine: MACE reduction (COLCOT, LoDoCo2); no cancer endpoint data Statins: MACE reduction + pleiotropic immune effects; observational cancer signal only	No trial powered for simultaneous CVD + oncologic endpoints No CHIP genotype‐stratified cancer prevention RCT No validated immune profiling protocol for pre‐ICI cardiovascular risk stratification

Abbreviations: CAC, coronary artery calcium; CHIP, clonal hematopoiesis of indeterminate potential; HSPC, hematopoietic stem and progenitor cell; ICI, immune checkpoint inhibitor; RCT, randomized controlled trial; sEV, small extracellular vesicle; TME, tumor microenvironment; VAF, variant allele frequency.

Translating this framework into clinical practice will require prospective cohort infrastructure capturing longitudinal immune, genomic, and imaging phenotypes across the cardio‐oncology continuum. Trial enrichment strategies based on CHIP genotype, inflammatory biomarker burden, immune checkpoint programs, and baseline vascular risk could identify patients with the highest dual inflammatory load and greatest potential for coordinated therapeutic benefit. Equally important will be dedicated interdisciplinary training that builds a community of investigators fluent across cardiology, oncology, immunology, genomics, and clinical trial design. The ambition of the next decade should not be merely to manage the cardiovascular complications of cancer therapy, but to therapeutically leverage shared immunological vulnerabilities in patients who bear the highest dual burden of heart disease and malignancy.

## Funding

This work was supported by the National Institutes of Health (R01HL153712, R35HL161185, R01HL172335, R01HL172365, P01HL131481), American Heart Association (26POST1563188), Fondation Leducq (20CVD02), DFG (HO 7496/1‐2).

## Conflicts of Interest

The authors declare no conflicts of interest.

## Data Availability

Data sharing not applicable to this article as no datasets were generated or analysed during the current study.
